# Serial follow-up of repeat voluntary blood donors reactive for anti-HCV ELISA

**DOI:** 10.4103/0973-6247.75979

**Published:** 2011-01

**Authors:** N. Choudhury, Sunita Tulsiani, Priti Desai, Ripal Shah, Ankit Mathur, V. Harimoorthy

**Affiliations:** *Department of Transfusion Medicine, Tata Medical Centre, Calcutta, India*; 1 *Jeevan Jyoti Blood Bank, Nagpur, India*; 2 *Tata Medical Centre, Mumbai, India*; 3 *Gujarat State Council for Blood Transfusion (GSCBT), Ahmedabad, India*; 4 *Ttk Rotary Blood Bank, Bangalore, India*; 5 *Prathama Blood Center, Vasna, Ahmedabad-380 007, India*

**Keywords:** Anti-HCV ELISA, repeat voluntary blood donor, occult infections, donor follow-up, nucleic acid test, recombinant immunoblot assay

## Abstract

**Background:**

Voluntary non-remunerated repeat blood donors are perceived to be safer than the first time blood donors. This study was planned for follow-up of previous hepatitis C virus (HCV) test results of anti-HCV enzyme-linked immunosorbent assay (ELISA) reactive repeat blood donors. The aim was to suggest a protocol for re-entry of the blood donors who are confirmed HCV negative by nucleic acid test (NAT) and recombinant immunoblot assay (RIBA). A group of repeat voluntary donors were followed retrospectively who became reactive on a cross sectional study and showed HCV reactivity while donating blood regularly.

**Material and Methods:**

A total of 51,023 voluntary non remunerated blood donors were screened for anti-HCV ELISA routinely. If anybody showed positivity, they were tested by two ELISA kits (screening and confirmatory) and then confirmed infection status by NAT and or RIBA. The previous HCV test results of repeat donors reactive by anti-HCV ELISA were looked back from the records. Data of donors who were repeat reactive with single ELISA kit (in the present study) were analyzed separately from those reactive with two ELISA kits (in the present study).

**Results:**

In this study, 140 (0.27%) donors who were reactive by anti HCV ELISA were included. Out of them, 35 were repeat voluntary donors and 16 (11.43%) were reactive with single ELISA kit. All 16 donors were reactive by single ELISA kit occasionally in previous donations. Their present ELISA positive donations were negative for HCV NAT and RIBA. A total of 19 (13.57%) donors were reactive with two ELISA kits. In their previous donations, the donors who were reactive even once with two ELISA kits were consistently reactive by the same two ELISA kits in their next donations also.

**Conclusion:**

Donor sample reactive by only single ELISA kit may not be considered as infectious for disposal as they were negative by NAT and or RIBA. One time ELISA positivity was found probably due to ELISA kit specificity and sensitivity. Donors reactive with two ELISA kit should be discarded as there is a high positivity with NAT/ RIBA. However, donors reactive by two ELISA kits and negative by NAT and RIBA should be followed up and may not be deferred permanently.

## Introduction

Safety and adequacy remain the central goal of donor screening programs.[1] Incidence rates for viral infections in blood donor may vary with the frequency of donation. It is observed that rate of transfusion transmitted infections is lower among repeat donors, especially in donors with higher frequency of donations.[2] Voluntary non-remunerated blood donors who give blood regularly are considered to be donors at the lowest risk of all because their blood is tested frequently.[3] However, a large number of eligible donors are lost because of apparently false-positive screening test results for transfusion transmitted infections (TTI). Notification of false-positive results to blood donors has been reported to cause psychological distress because the cause and the clinical significance of these results are often not known.[4] This prospective study was planned to look back at the hepatitis C virus (HCV) test results of repeat donors reactive by anti-HCV enzyme-linked immunosorbent assay (ELISA). The results were corroborated with previous donation record of the same donor in the same blood center to suggest a protocol for re-entry of the blood donors who were confirmed HCV negative by nucleic acid test (NAT) and recombinant immunoblot assay (RIBA).

## Material and Methods

The present study was conducted at a regional blood transfusion center (Prathama Blood Center, Ahmedabad) in western India for a total period of 15 months from August 2007 to October 2008. A total of 51,023 donors were screened for anti-HCV by ELISA in the process of routine screening by a single kit. The donor samples which were initially reactive for anti-HCV were tested in duplicate by the same ELISA kit. When either of the samples was reactive in duplicate testing, the same was tested by a second ELISA kit. All the donor samples reactive by either first or the second ELISA kit were then tested by NAT and/or by RIBA. All samples were tested by NAT and NAT negative samples were again tested by RIBA. Test results of repeat donors were studied and the previous ELISA results of the same repeat donors were analyzed. As a policy, all TTI positive blood donors were informed with pre- donation counseling as per standard procedures. However, these blood donors donated blood inspite of them being informed of the TTI status. There was no system to detect TTI status of blood donors before collection especially during outdoor camps as the whole process was not computerized. However, all units showing reactive results in that particular donation were discarded as per standard procedures.

All blood donors with discordant result were followed up prospectively for further analysis. The previous anti-HCV results of the repeat donors were looked back from the donation record and ELISA testing record. Data of donors who were repeat reactive in single ELISA kit (in the present study) were analyzed separately from those reactive in two ELISA kits (in the present study). During the study period, two approved ELISA kits were used (kit A and kit B). In a few instances, another kit (kit C) was also used in the case of non-availability of either kit. Each donation was tested either by kit A or B and once it was positive, the sample testing was repeated by the other ELSA kit. For each donor, the number of donation, testing kit in which the test was reactive, and results of previous donations when tested with kit A, kit B and or kit C were mentioned. ELISA kits used during the study period were Murex version 4, Hepanostika Ultra HCV and LG-HCV.

## Results

### Repeat blood donors

In the study group, 105 (75%) donors were first time donors and 35 (25%) donors were repeat donors [Table 1]. Among the repeat donors, 16 (11.43%) were reactive in single ELISA kit and 19 (13.57%) were reactive in two ELISA kits. Details of the previous donations are given in Tables 2 and 3. Table 2 describes the details of repeat donors reactive in single ELISA kit. Table 3 describes the details of repeat donors reactive in two ELISA kits. If any sample was reactive by more than one ELISA kit, names of both the kits were recorded. Results of NAT and RIBA are also given in the both tables.

**Table 1:** First time and repeat donors in the study group

First time donors (%)	Repeat donors (%) 35 (25)		Total donors
105 (75)	Repeat Single kit reactive: 16 (11.43)	Repeat two kits reactive: 19 (13.57)	140

N.B.- Initially, 165 donor samples were collected. However, only 140 donor samples which met the inclusion criteria were included in the study

**Table 2:** Repeat donors reactive in single ELISA kit

Donor no.	Present donation		Previous donations Results			Present donation	
	No. of donation when reactive	Name of kit	Kit A	Kit B	Kit C	NAT	RIBA
14	5^th^	Kit B	NR* (1^st^, 2^nd^, 3^rd^ D)	RE (4^th^ D)	-	N	N
15	2^nd^	Kit B	NR (1^st^ D)	-	-	N	N
27	4^th^	Kit B	NR (1^st^, 2^nd^ D)	NR (3^rd^ D)	-	N	N
47	2^nd^	Kit B	NR (1^st^ D)	-	-	N	N
50	2^nd^	Kit A	-	NR (1^st^ D)	-	N	N
57	15^th^	Kit B	NR (10^th^, 13^th^ D)	NR (12^th^ D)	NR (1^st^–9^th^, 11^th^, 14^th^ D)	N	N
60	3^rd^	Kit A	NR (2^nd^ D)	NR (1^st^ D)		N	N
62	2^nd^	Kit A	-	NR (1^st^ D)		N	N
75	3^rd^	Kit A	-	NR (2^nd^ D)	NR (1^st^ D)	N	N
80	2^nd^	Kit A	NR (1^st^ D)	-	-	N	N
82	2^nd^	Kit A	NR (1^st^ D)			N	N
87	2^nd^	Kit A	-	-	NR (1^st^ D)	N	N
88	4^th^	Kit A	RE (3^rd^ D)	NR (1^st^, 2^nd^ D)	-	N	N
90	2^nd^	Kit A	-	-	NR (1^st^ D)	N	N
114	4^th^	Kit B	-	NR (2^nd^, 3^rd^ D)	NR (1^st^ D)	N	N
165	6^th^	Kit A	-	-	NR (1^st^–5^th^ D)	N	N

* NR= nonreactive

RE= reactive; ‘-’= not tested; D= donation; NEG= negative; Pos= positive; IND= indeterminate, N.B: All repeat donors reactive in single kit were negative for NAT and RIBA. They were reactive by a particular kit in the present donation but non-reactive by other kits in previous donations.

**Table 3:** Repeat donors reactive in two ELISA kits

Donor no.	Present donation	Previous donations Results				Present donation	
	No. of donation when reactive (both Kit A/B)	Kit A	Kit B	Both Kit A /B	Kit C	NAT	RIBA
3	2^nd^	NR* (1^st^ D)	-	-	-	NEG	IND
17	5^th^	NR (1^st^ D)	NR (3^rd^ D)	-	NR (2^nd^, 4^th^ D)	NEG	NEG
20	2^nd^	-	RE (1^st^ D)	-	-	NEG	IND
36	4^th^	NR (1^st^ D)	NR (2^nd^ D)	-	-	NEG	NEG
			RE (3^rd^ D)				
41	3^rd^	NR (2^nd^ D)	-	-	NR (1^st^ D)	NEG	IND
48	4^th^	-	NR (2^nd^)	RE (3^rd^ D)	NR (1^st^ D)	NEG	POS
51	3^rd^			RE (1^st^, 2^nd^ D)	-	POS	-
68	2^nd^	NR (1^st^ D)	-	-	-	NEG	IND
73	3^rd^	-	NR (2^nd^ D)	-	NR (1^st^ D)	POS	-
76	2^nd^	-	NR (1^st^ D)	-	-	NEG	IND
79	2^nd^	-	-	-	NR (1^st^ D)	NEG	POS
99	2^nd^	RE (1^st^ D)	-	-	-	NEG	NEG
115	4^th^	-	NR (1^st^ D)	-	NR (2^nd^, 3^rd^ D)	NEG	NEG
123	3^rd^	-	-	RE (1^st^, 2^nd^ D)	-	POS	-
131	11^th^	NR (4^th^, 8^th^, 9^th^,	-	-	NR (1^st^, 2^nd^, 3^rd^, 5^th^,	POS	-
		10^th^ D)			6^th^, 7^th^ D)		
140	2^nd^	-	RE (1^st^ D)	-	-	NEG	IND
144	2^nd^	-	NR (1^st^ D)	-	-	NEG	IND
145	2^nd^	NR (1^st^ D)	-	-	-	NEG	POS
157	2^nd^	-	-	RE (1^st^ D)	-	NEG	POS

* NR= nonreactive

RE= reactive; ‘-’= not tested; D= donation; NEG= negative; Pos= positive; IND= indeterminate, N.B: All repeat donors reactive in single kit were negative for NAT and RIBA. They were reactive by a particular kit in the present donation but non-reactive by other kits in previous donations

### Blood donors reactive with single ELISA kit only

Among the repeat donors, 16 (11.43%) donors were reactive with single ELISA kit in the present study. The results of their previous donations were analyzed. All these donors were at least once nonreactive by ELISA. In subsequent donations, they became reactive by ELISA. NAT and RIBA were negative in all repeat donors who were reactive only by single ELISA kits.

#### Donors with special observations

Donor No. 14: It is evident from serial testing record that this donor was repeatedly reactive by kit B two times. The same donor was always nonreactive by the other kit (kit A) three times. The 5^th^ donation sample was included in the study and was negative by NAT and RIBA. After this 5^th^ donation (study sample) also, the donor was followed prospectively and had donated twice. The results remained reactive by kit B (6^th^ donation) and nonreactive by kit A (7^th^ donation). One important point was that over a period of 27 months, the results of kit A and also kit B remained consistent throughout.

Donor no. 27: This donor’s sample was nonreactive twice with kit A. The same donor’s sample was nonreactive by kit B in the previous donation but reactive in the present donation by kit B (study sample). However, the sample was negative by NAT and RIBA.

Donor no. 57: This donor had donated 15 times. This donor was nonreactive 14 times by all the three kits in different occasions. The present study sample (15^th^ donation) was reactive by ELISA (kit B), but was negative by NAT and RIBA. This donor donated once again (16^th^ donation) and was tested again nonreactive by kit A.

Donor no. 75: This donor’s sample was nonreactive in two donations by two different kits (kit B and kit C). In the present donation, i.e. 3^rd^ donation, the sample was reactive by kit A, but the NAT and RIBA results were negative.

Donor no. 88: This donor had donated total four times. In the first two donations, the sample was nonreactive by kit B. Third and fourth donation samples were reactive by kit A only. The 4^th^ donation sample was included in the study group and was negative by NAT and RIBA.

Donor no. 114: This donor was negative three times by two different kits (kit B or kit C). The present sample (4^th^ donation) was reactive by kit B but negative by NAT and RIBA.

Donor no. 165: This donor was negative by kit C five times. In the 6^th^ donation, the donor was found to be reactive by kit A only. NAT and RIBA results were negative for this donation

### Blood donors reactive with two ELISA kits

Among the repeat donors, 19 (13.57%) samples were reactive with two ELISA kits in the study group sample. Few of these samples were reactive by both kit A and kit B in the previous donations also. The results of their previous donations were analyzed for all the 19 donors. Out of 19 donors, 8 (42.1%) were confirmed positive by NAT or RIBA.

#### Donors with special observations:

Donor no.17: Donor was nonreactive by kit B or kit C till the 4^th^ donation. On 5^th^ donation, sample (study group) was reactive by kit A. This donor was also negative by NAT and RIBA.

Donor no. 20: Donor was nonreactive by kit B in the 1^st^ donation, but was reactive by both the kits in the 2^nd^ donation (study sample). This sample was negative by NAT and indeterminate by RIBA. No history suggestive of HCV associated risk factor was revealed by the donor at the time of blood donation. Serious efforts were made to follow up this donor however; the donor was lost to follow up.

Donor no. 48: Sample was nonreactive by kit C in the 1^st^ and by kit B in the 2^nd^ donation sample. The 3^rd^ donation sample was reactive by kits A and B. The present donation (4^th^) was reactive by two kits and was included in the study. This sample was NAT negative but RIBA positive. The donor did not mention any HCV associated risk factor history at the time of donation and history of cirrhotic liver in the family.

Donor no. 51: Donor was reactive by kit A and kit B in the 1^st^ to 3^rd^ donation. Third donation was included in the study group and was NAT positive. This donor was asymptomatic but revealed history of blood transfusion about 10 years back.

Donor no.73: This donor was negative by kit C once and by kit B the other time. In the third donation, the donor was reactive by both the kits. This sample was found to be NAT positive. This donor also did not reveal any suggestive history related to risk factor for HCV at the time of donation. This donor could not be followed up.

Donor no. 115: Donor was nonreactive three times either by kit B or kit C. The 4^th^ donation was reactive by both the kits, but negative by NAT and RIBA.

Donor no. 123: This donor was reactive by both kits A and B in all the three donations. The 3^rd^ sample (study group) was positive by NAT. No HCV risk factor associated history was revealed at the time of donation.

Sample no. 131: Donor had donated blood 11 times. Sample was reactive by kit C from 1^st^ to 3^rd^ and from 5^th^ to 7^th^ donation. The sample was nonreactive in the 4^th^ and from 8^th^ to 10^th^ donation by kit A. In the 11^th^ donation (study sample), it was reactive by kits A and B both. This sample was also NAT positive. No history of association of risk factor for HCV was known for this donor.

Donor no. 157: Reactive by both the kits in the first donation. The second donation was included in the study group and was reactive by both the kits. The sample was NAT negative and RIBA positive. This donor also had not mentioned any HCV risk associated history at the time of blood donation.

In donors whose samples were reactive even once with two kits, were consistently reactive by the same two kits in their next donations also. All donors who were previously reactive by two ELISA kits were informed about their HCV status through a letter. These donors were counseled to consult a physician and not to donate again. This is done as a part of standard operating procedure for post-donation counseling of TTI reactive donors. In spite of this, these donors had donated again. Though the software had a provision of making these donor data inactive so that they were not called for blood donation, there was no identification system at donation sites and blood donation camps. Few of these donors had donated against the medical advice, without the knowledge of blood center.

## Discussion

Data of donors who were repeat reactive with single ELISA kit (in the present study) were analyzed separately from those reactive with two ELISA kits (in the present study).

### Donors reactive in only single ELISA kit:

It was observed during follow-up of repeat blood donors that donors reactive by only single ELISA kit were repeatedly reactive by the same kit and nonreactive by the other kit. These donors were all NAT and RIBA negative. As observed in sample no. 14, this sample was consistently nonreactive by kit A and was reactive by kit B. Most probably, the same sample had some cross-reactivity with some antigens in the ELISA kit B and was consistently reactive with it. The donor of sample no. 57 had donated blood 16 times. The 15^th^ donation sample was reactive by kit B. This sample was negative by NAT and RIBA, but this blood unit would have been discarded on the basis of test done by kit B. Donor no. 88 donated blood totally four times. This donor’s sample was also consistently nonreactive two times by kit B and twice reactive by kit A. The sample was negative by NAT and RIBA. It was evident from the test results of above donors that samples which were reactive with only single ELISA kit may not be confirmed ELISA positive. These samples give consistent reactive result with a particular kit and nonreactive result with the other kit. These samples might have some cross-reactivity with some antigen in the kit. As stated by Sharma *et al*., false-positive test results in otherwise healthy blood donors have been attributed to the presence of cross-reacting circulating antigens and antibodies. Several factors may be associated with screening test reactive but confirmatory test negative and intermediate results. A history of allergy, acute illness, or alloimmunization, the presence of autoantibodies, or vaccination may result in false-reactive test results.[4]

These blood units which were reactive by single kit only were always discarded as per existing law. These were actually negative samples when tested by NAT or RIBA and were not infectious. These donations were unnecessarily discarded. Similar findings were reported in a study conducted in China by Ren *et al*., in which 156 samples were tested by seven ELISA kits and those with discrepant results in the different kits were negative by both NAT and RIBA.[5]

### Donors reactive by two ELISA kits

Out of 19 donors, 8 had confirmed HCV infection demonstrated by positive NAT or RIBA results. It was observed in sample no. 17 which was nonreactive by any of the kits (A or B or C) till the present donation. In the study group sample, the sample was reactive with both the kits, but was negative by NAT and RIBA. Sample no. 48 was reactive with two kits in the 3^rd^ and 4^th^ donation. The 4^th^ donation sample was negative by NAT but RIBA positive, which might be because the donor HCV RNA level was below the detection level but the antibody level remained elevated. This incident justifies that blood donor sample should be tested and reconfirmed by two ELISA kits before discarding the unit and not based on the result of single ELISA result. As observed by Seed *et al*., two assays applied sequentially can increase the positive predictive value of the process by selecting for true positive reaction because samples reactive in both the assays have a higher probability of representing true reaction rather than those reacting only in one. World Health Organization (WHO) guidelines also (2002) mentioned that if confirmatory testing is not available, use an alternate assay that is as sensitive as the primary assay, for use in confirming the status of the samples that are found to be repeatedly reactive by the primary assay.[3,6]

Sample nos. 79, 145 and 157 were also negative by NAT but RIBA was positive. The reason may be that the circulating HCV RNA titer may vary considerably. While a single qualitative assay for HCV RNA confirms active viral replication, a single negative test does not exclude viremia and may reflect only a viral load below the detection limit of the assay.[7] So, the Center for Disease Control (CDC), Atlanta has mentioned that the significance of a single negative HCV RNA result is unknown, and the need for further medical evaluation is determined by verifying anti-HCV status.[8]

Donor no. 51 was reactive by both the kits in three donations and the last sample was NAT positive. This donor had history of blood transfusion in the past. In this donor, the results could be correlated with significant history of association of risk factor. Few other studies also have shown an association of history of blood transfusion with an increased risk of polymerase chain reaction (PCR) positivity or confirmed HCV cases.[9,10,11]

Donor no. 73 was nonreactive in first two donations. In the 3^rd^ donation, ELISA was reactive with two kits and also NAT positive. Most probably, the donor acquired infection during this interval between the 2^nd^ and 3^rd^ donation. The donor did not reveal any history of risk behavior during this period. It was assumed that this donor was a dangerous type of donor harboring occult infection. The donor was nonreactive by kit B and kit C in previous donations. However, in the 3^rd^ donation, the donor was reactive by both kits B and A. An attempt was made to trace the recipients of both these units without any success. Donor no. 115 was reactive by both the kits in the 4^th^ donation, but was NAT and RIBA negative. Such donors should be dealt carefully and regular follow-up should be done. They may not be deferred permanently. Sample no. 131 was nonreactive 10 times with single kit. The 11^th^ donation was reactive by kit A and B and this sample was also NAT positive. Again, this case was a highly dangerous case with occult infection. Due to unknown reason, probably due to low anti HCV antibody level, infection may not have detected for the last 10 times. and HCV viremia was confirmed by NAT on 11^th^ donation. This donor might have acquired infection before this donation but no such history could be elicited. Though this donor is a repeat donor and had donated 11 times, still was not safe as a donor. So, it is very important to take proper medical and risk behavior associated history at the time of donation. In this type of cases, necessity of blood donor screening by using NAT is firmly established. NAT for HCV was implemented by blood centers in 1999 in the United States. In 3 years of NAT testing, 170 HCV NAT-positive, seronegative donations were identified in the United States among 39.7 million screened donations. NAT has probably reduced the residual risk for HCV transmission to less than 1 in 2,000,000 components transfused.[12,13] In a study conducted in France in 2004, residual risks without NAT were estimated at 1 in 1,000,000 for HCV. With minipool NAT, the residual risk became nearly seven times lower for HCV, i.e. approximately 1 in 6.65 million donations.[14]

Donors who were ELISA reactive by two ELISA kits were informed about their HCV status and counseled not to donate again. In spite of this, these donors had donated again as there was no system of tracking by the software just before donation. The blood center did not have any method to check these donors and block them at the screening site before blood donation, especially at outdoor camp sites. So, these donors repeatedly donated blood without the knowledge of the blood center. If by chance, these donors’ sample is tested by a kit which is from different manufacturers, the blood unit may be labeled as nonreactive and issued to the patient. So, a provision in the blood bank software should be developed so that it can identify any donor whose sample is repeat reactive by two ELISA kits previously in the blood bank. There should be at least two identification marks for the donor i.e. date of birth or mother’s name or any other mark. The ideal situation will be, if with the help of this software, the donor is identified at the screening level only. But this may not be practical in most of the blood center setups in developing countries like India where most of Indian blood banks documentations are maintained manually. In such a case, the software should at least be able to identify the blood donor and block the blood unit and ensure that it is not issued to the patient.

Discordant results on regular voluntary blood donors in this study is a matter of concern and justified employment of NAT test on routine blood donor screening. It is evident that only dependence of repeat voluntary blood donor as a source of safe blood may not be sufficient in this part of the world. It may be due to many reasons like occult infections, non-complaint donors or contacting infections in between donations. However, cost benefit issues to be examined before taking further steps in implementation in any services.

Among the 19 donors who were reactive by two ELISA kits, 11 were negative or indeterminate by NAT and RIBA. These donors had not been reactive by two kits previously. These donors should be followed up at regular intervals. A protocol for re-entry of such donors is recommended. It was observed in a look-back study by Vrielink *et al*.[15] that none of the recipient’s of blood products from previous donations of anti-HCV ELISA positive, cDNA-PCR negative and RIBA 2 indeterminate or negative were HCV infected. Such donors were not infected and the author had suggested that these donors could re-enter the donor pool, provided that future donations were anti-HCV ELISA negative. In a study by Moore *et al*.[16], those donors whose samples were reactive both in the routine screening test and in the alternate assay were not withdrawn permanently from donation, but removed from the donor panel for an arbitrary period of 3 years. However, donors reactive by two ELISA kits and positive by NAT or RIBA should be permanently deferred from donating blood and should be advised for medical treatment.

## Conclusion

This study demonstrated that blood units from repeat donors reactive by single ELISA kit only (and nonreactive by the second ELISA kit) may not be discarded though this policy needs regulatory approval in respective countries. Repeat donors are perceived to be safe donors. However, no safety features like proper detailed history, screening and follow up etc, should be lowered only being the repeat donors. Donors reactive by two ELISA kits and positive by NAT or RIBA should be informed and deferred permanently. These donors should be advised to take medical treatment and instructed never to donate again. Donors reactive by two ELISA kits but negative by NAT and RIBA should be properly counseled. A protocol for re-entry of these donors may be made after long term follow up. A safety features to be included in all blood bank software which identify a previously two ELISA kit reactive blood donor and blocks the blood unit from issue.

## References

[1] Stramer SL (2006). Impact of NAT on serological screening of transfusion transmitted agents. ISBT Science Series.

[2] Schreiber GB, Glynn SA, Busch MP, Sharma UK, Wright DJ, Kleinmann SH (2001). Incidence rates of viral infections among repeat donors: are frequent donors safer?. Transfusion.

[3] (2002). Anonymous. Safe Blood Donation, In Safe Blood and Blood products: Identifying low risk donors.

[4] Sharma UK, Stramer SL, Wright DJ, Glynn SA, Hermansen S, Chreiber GB (2003). Impact of changes in viral marker screening assays. Transfusion.

[5] Ren FR, Lv QS, zHuang H, Li JJ, Gong XY, Gao GJ (2005). Significance of the signal-to –cut off ratios of anti-hepatitis C virus enzyme. Transfusion.

[6] Seed CR, Margaritis AR, Bolton WV, Kiely P, ParkerS, Piscitelli L (2003). Improved efficiency of national HIV, HCV and HTLV antibody testing algorithms based on sequential screening immunoassays. Transfusion.

[7] Molin GD, Tiribelli C, Campello C (2003). A rational use of laboratory tests in the diagnosis and management of hepatitis C virus infection. Ann Hepatol.

[8] Alter MJ, Kuhnert WL, Finelli LF (2003). Centers for Disease Control and Prevention: Guidelines for laboratory testing and result reporting of antibody to hepatitis C virus. MMWR Recomm Rep.

[9] Kim YS, Lee HS, Ahn YO (1999). Factors associated with positive predictability of Anti-HCV ELISA method with confirmatory RT-PCR. J Korean Med Sci.

[10] Qui Y, Shi L, Wang Y, Zhang G, Zheng J, Gong X (2008). Risk factors for hepatitis C virus infection among blood donors in Beijing and implications for improving the pretesting donor screening process. Transfusion.

[11] Tucker TJ, Voigt M, Bird A, Robson S, Gibbs B, Kanneyer J (1997). Hepatitis C virus infection rate in volunteer blood donors from the Western Cape- comparison of screening tests and PCR. S Afr Med J.

[12] Goodnough LT, Shander A, Brecher ME (2003). Looking to the future. Transfus Med.

[13] Busch MP (2005). A new strategy for estimating risks of transfusion- transmitted viral infections based on rates of detection of recently infected donors. Transfusion.

[14] Pillonel J, Laperchi S (2004). Trends in residual risk of transfusion transmitted viral infections (HIV, HCV, HBV) in France between 1992 and 2002 and impact of viral genome screening (Nucleic Acid Tesing). Transfus Clin Biol.

[15] Vrielink H, Reésink HW, Zaaijer HL, Scholten E, Kremer LC, Cuypers HT (1997). Look back of anti-HCV ELISA positive, HCV-RNA PCR-negative donors and recipients of their blood products. Vox Sang.

[16] Moore MC, Howell DR, Barbara JA (2007). Donors whose blood reacts falsely positive in transfusion microbiology screening assays need not be lost to transfusion. Transfus Med.

